# Association of dietary habits and physical activity with the severity of fecal incontinence in patients with colorectal cancer after minimally invasive surgery: An exploratory study

**DOI:** 10.1016/j.apjon.2026.100953

**Published:** 2026-04-09

**Authors:** Hiromi Nakagawa, Sho Hatanaka, Nobuhisa Matsuhashi, Hiroyuki Sasai

**Affiliations:** a Department of Nursing, Gifu University School of Medicine, Gifu, Japan; b Research Team for Promoting Independence and Mental Health, Tokyo Metropolitan Institute for Geriatrics and Gerontology, Tokyo, Japan; c Department of Gastroenterological surgery and Pediatric surgery, Surgical Oncology, Gifu University School of Medicine, Gifu, Japan

**Keywords:** Colorectal cancer, Survivors, Fecal incontinence, Physical activity, Sedentary behavior

## Abstract

**Objective:**

Fecal incontinence (FI) is a common and distressing long-term complication following minimally invasive surgery (MIS) for colorectal cancer (CRC), yet the role of modifiable lifestyle factors remains unclear. This study examined the associations between dietary habits (red meat, fiber-rich foods, sugar-sweetened beverages, and alcohol), physical activity (PA) duration, and sedentary behavior with FI severity in CRC patients after MIS.

**Methods:**

This exploratory cross-sectional study recruited CRC patients attending a university hospital outpatient clinic in Japan. Dietary habits and PA were assessed via self-administered questionnaires; FI severity was measured using the Wexner score. Multiple linear regression models adjusted for age, sex, tumor location, chemotherapy use, laxative use, and postoperative years were fitted for each exposure.

**Results:**

A total of 136 patients completed the questionnaire. Participants had a median age of 72 years and 50% were women; 131 (96.3%) reported FI (mean 1.9 ± 0.9 episodes/day). Higher red meat intake (≥ 500 g/week) was associated with lower FI severity (*β* = −1.42; 95% confidence interval [CI], −2.71 to −0.12), whereas prolonged sedentary time (≥ 8 h/day) was associated with higher FI severity (*β* = 1.36; 95% CI, 0.18 to 2.54). PA duration was not associated with FI severity.

**Conclusions:**

Red meat intake and sedentary behavior were associated with FI severity. These associations should be interpreted cautiously; longitudinal and interventional studies are warranted to clarify causality.

## Introduction

Colorectal cancer (CRC) is the third most common cancer worldwide.^1^ Postoperative bowel dysfunction, particularly fecal incontinence (FI), is a common long-term complication after colorectal surgery and can substantially impair patients’ quality of life (QoL). The prevalence of liquid and solid FI following standard colon surgery has been reported to be 24.1% and 6.9%, respectively. According to the International Continence Society, FI is defined as the involuntary loss of solid or liquid feces.^2^ Rectal surgery may damage the internal anal sphincter, particularly during intersphincteric resection, often resulting in FI.^3^ Although minimally invasive surgery (MIS), including laparoscopic and robot-assisted procedures, improves postoperative recovery compared with conventional open surgery, persistent bowel dysfunction, including FI, remains common.^4^ Postoperative FI is known to impair QoL,^5^ and long-term follow-up studies have reported that 46%–49% of patients undergoing sphincter-preserving surgery develop defecation disorders, including FI.^6^

Treatment options for FI include antidiarrheal drugs, pelvic floor muscle training, biofeedback therapy, and surgical procedures.^4^ However, these interventions often have limited efficacy, and access to such treatments may be uneven. Consequently, lifestyle-based management strategies have attracted increasing attention, particularly dietary modification and physical activity (PA), as non-invasive approaches to improve bowel function.^5^ Non-surgical approaches, such as optimizing stool consistency, dietary modification, and pelvic floor muscle training, may reduce FI severity, although supporting evidence remains limited.^7^^,^^8^

Several studies suggest that dietary factors may influence bowel function and FI. A prospective cohort study of 58,330 older American women found a lower incidence of FI among those consuming at least 25 g of dietary fiber per day,^9^ suggesting a potential protective effect of fiber intake. Fiber-rich dietary patterns have also been associated with increased bowel movement frequency^10^ and may influence bowel habits and QoL in CRC survivors.^11^ In patients undergoing sphincter-preserving rectal surgery, moderate meat intake has been associated with improved postoperative bowel function, including reduced FI.^12^^,^^13^ Sugar-sweetened beverages may cause osmotic diarrhea and bloating due to colonic fermentation,^14^ whereas alcohol intake may disrupt gut microbiota diversity, alter gastrointestinal motility, and increase intestinal mucosal permeability.^15^ Overall, dietary habits may influence bowel symptoms, although evidence in CRC survivors remains inconsistent.

PA and sedentary behavior may also influence pelvic floor function and bowel control. Insufficient PA and prolonged sedentary behavior have been associated with pelvic floor dysfunction, including FI.^16^ After cancer treatment, patients with CRC tend to have lower PA levels^17^ and spend more time sedentary than the general population.^18^ Pelvic floor dysfunction is a major cause of FI because impaired support of the rectum and sphincter complex may result in loss of bowel control.^19^ Prolonged sedentary behavior may further impair intestinal motility and pelvic floor muscle function.^20^ Therefore, maintaining sufficient PA and reducing sedentary time may support overall physical function^21^ and potentially pelvic floor-related function. However, although PA and sedentary behavior have been investigated in CRC populations, their associations with FI severity remain unclear.^16^

Dietary habits and PA represent modifiable lifestyle factors that may influence bowel function. However, the associations between these lifestyle factors and FI severity in patients with CRC after MIS remain unclear. Therefore, the present exploratory study aimed to examine the associations between dietary habits, including red meat, fiber-rich foods, sugar-sweetened beverages, and alcohol consumption, and PA-related factors, including PA duration and sedentary behavior, and FI severity in patients with CRC after MIS.

## Methods

### Study design, participants, and setting

This exploratory cross-sectional study was conducted at a tertiary university hospital in Japan. Adults aged 20 years or older with a history of CRC who visited the outpatient gastrointestinal surgery department were recruited between February and May 2024.

The age cutoff of ≥ 20 years was applied primarily for clinical and methodological reasons related to the adult care context. This study was conducted within the framework of adult clinical care, and pediatric and adolescent patients were excluded because their disease characteristics, treatment strategies, postoperative courses, and lifestyle behaviors differ markedly from those of adults. Including these age groups could have introduced clinical heterogeneity and compromised the internal validity of the analysis. In addition, lifestyle-related factors such as alcohol consumption were assessed in this study. As alcohol consumption is legally permitted from the age of 20 in Japan, this criterion ensured consistency and validity in evaluating lifestyle behaviors.

Patients who met the eligibility criteria and expressed interest in participation during their outpatient visit were referred by their physicians to the research office, where eligibility was confirmed and the study procedures were explained. Written informed consent was obtained from all participants prior to enrollment. A convenience sample was used, consisting of all eligible patients who received follow-up care at the hospital during the study period and met the inclusion criteria.

Eligibility criteria included a history of MIS for CRC within the past 10 years and the availability of surgical records. The 10-year postoperative period was chosen as a clinically relevant timeframe, as prior studies suggest this is sufficient for evaluating the development of chronic FI. In this study, MIS was defined as robotic or laparoscopic surgery designed to minimize surgical trauma.^22^

Exclusion criteria included a prior or current diagnosis of local recurrence, mental illness, severe arrhythmia, dementia, prior or ongoing radiotherapy, and current dialysis. Patients who had undergone radiotherapy were excluded to minimize the influence of radiation-induced bowel dysfunction and to focus on FI primarily attributable to surgical factors. Additionally, patients with a stoma were excluded because FI was assessed via the anal route.

The study was conducted using a self-administered multiple-choice questionnaire.

To ensure data completeness and reduce the risk of missing data or post-completion modification, all questionnaires were distributed and collected in person at the hospital.

Participants were provided with assistance as needed to ensure accurate understanding of questionnaire items and appropriate completion, particularly given the older age of the study population. After completion, each questionnaire was reviewed by research staff during a face-to-face interview to confirm clarity and completeness. Any missing or unclear responses were clarified immediately. Questionnaires were not considered complete until all items had been checked and confirmed.

To minimize potential information bias and recall bias, the questionnaire consisted of structured multiple-choice items with predefined response options. In addition, standardized examples of commonly consumed foods and reference portion sizes were provided to facilitate consistent estimation of dietary intake, and research staff ensured that responses reflected participants’ usual habits without leading or prompting specific answers.

Once verified, the questionnaires were securely stored in a locked cabinet accessible only to the research team. No responses were altered after verification.

### Patient characteristics

Patient characteristics included sex, age, body mass index (BMI), household type (living alone or with others), employment status, tumor-node-metastasis (TNM) stage according to the Union for International Cancer Control (UICC), operative procedure, and relevant medical history (such as diabetes mellitus, hypertension, angina pectoris, or respiratory diseases). None of the participants with stage IV or an unknown stage were receiving active cancer treatment at the time of the survey.

### Dietary habits

We assessed four dietary components, red meat, fiber-rich foods, sugar-sweetened beverages, and alcohol consumption, based on dietary guidelines from the American Cancer Society (ACS),^23^ the World Cancer Research Fund (WCRF) and the American Institute for Cancer Research (AICR).^24^ These items were selected because they are consistently highlighted in international guidelines for cancer prevention.

Red meat intake was assessed using the question “How many grams of red meat do you consume per week?” The red meat was of any type, regardless of processing. Based on WCRF/AICR recommendations,^24^ intake was categorized as < 500 g or ≥ 500 g per week.

Fiber-rich food intake was assessed using the question “How many grams of fiber-rich foods do you consume per day?” In this study, fiber intake referred to the total amount derived from naturally fiber-rich foods, including whole grains, fruits, vegetables, and legumes.^24^ Although the WCRF/AICR recommends a combined daily intake of at least 400 g/day of fruits and non-starchy vegetables,^24^ we used 200 g/day as a cut-off to represent a level substantially below the recommended standard.

Sugar-sweetened beverage intake was assessed using the question “How many grams of sweetened beverages do you consume per day?” Based on the same dietary guidelines,^24^ intakes were categorized as < 250 or ≥ 250 g/day.

Alcohol consumption was assessed using the question “How many grams of alcohol do you consume per day?” Alcohol consumption was assessed based on total ethanol intake in accordance with the 2018 WCRF/AICR scoring standards. Following cancer prevention guidelines,^24^ alcohol consumption was classified as < 28 g/day or ≥ 28 g/day (ethanol).

### Physical activity and sedentary behavior

PA and sedentary behavior were assessed using the Global Physical Activity Questionnaire (GPAQ),^25^ a validated instrument developed by the World Health Organization to measure PA across diverse populations. The instrument assesses three domains: occupational, transportation, and recreational activities. It offers a comprehensive overview of participants' daily activity levels. The GPAQ requires respondents to report PA episodes lasting at least 10 minutes.^26^ Total PA was considered a primary exposure variable, while domain-specific PA (occupational, transportation, and recreational) and sedentary behavior were treated as secondary exposure variables. Occupational PA included employment, volunteer work, part-time jobs, academic activity, housework, caregiving, agricultural work, fishing, job seeking, and other chores. In this study, the cutoff for total PA was set at ≥ 300 min/week,^27^ and that for each domain-specific PA was set at ≥ 100 min/week. As no standard cut-off exists for domain-specific PA, 100 min/week was adopted by dividing 300 min by the number of domains. Sedentary behavior was defined as “all waking behaviors performed in a sitting or lying position with an energy expenditure of ≤ 1.5 metabolic equivalents”^26^ and the cutoff for prolonged sedentary behavior was set at ≥ 8 h/day.^28^

Cutoff values were selected to facilitate clinical interpretability for health care professionals and to align with guideline-based recommendations for cancer survivors. Hence, PA and sedentary behavior were analyzed as meaningful behavioral categories rather than continuous dose–response variables.

### Severity of fecal incontinence

The severity of FI, investigated as the primary outcome, was assessed using the Wexner score.^29^ The Wexner score is calculated from the responses to five questions in the following categories: gas, solid stool, liquid stool, use of a pad, and lifestyle alteration. Each item is scored on a scale of 0–4, yielding a total score of 0–20 points. Higher scores indicate greater FI severity, with the following categories: no FI (0 points), mild FI (1–4 points), moderate FI (5–8 points), severe FI (9–12 points), highly severe FI (13–16 points), and extremely severe FI (17–20 points), characterized by persistent symptoms that significantly impair daily life and QoL.^30^ Additionally, to evaluate FI, we collected data on the mean number of incontinence episodes per day.

### Statistical analysis

A formal sample size calculation was not performed due to the exploratory nature of this study, and the sample size was determined by the number of eligible patients during the study period. Therefore, the study may have been underpowered to detect modest associations, and the findings should be interpreted as hypothesis-generating. Given the relatively small sample size and model constraints to avoid overfitting, non-significant findings should be interpreted with caution.

Descriptive statistics are presented as *n* (%), mean (standard deviation [SD]), or median (interquartile range [IQR]), depending on the type and distribution of data.

All exposure variables, including dietary exposures and PA, were treated as categorical variables using predefined cutoffs based on established international guidelines (e.g., WCRF/AICR^24^ for dietary factors and World Health Organization [WHO]^26^ for PA). These cutoffs are widely used in cancer survivorship research. They were considered appropriate for this study population and were used to enhance clinical interpretability and facilitate comparison with previous studies.

Before categorization, we examined the distributions of continuous variables and their relationships with FI severity to ensure that the selected cutoffs were reasonable and that no strong deviations from linearity were observed.

Dietary exposures included red meat, fiber-rich foods, sugar-sweetened beverages, and alcohol consumption, while PA-related exposures included total PA and sedentary behavior.

We used multiple linear regression models to examine associations between each exposure variable and FI severity, as measured by the Wexner score.

Each lifestyle-related exposure was entered into a separate regression model together with covariates including sex, age, chemotherapy use (yes or no), laxative use (yes or no), tumor location (rectum or colon), and postoperative years (< 2 years or ≥ 2 years), which were selected based on prior literature and clinical relevance.^31^ This modeling strategy was adopted primarily due to the relatively small sample size and to reduce the risk of model overfitting. Including multiple correlated lifestyle variables in a single model may introduce multicollinearity and compromise model stability; therefore, separate models were used. As a result, the independent effects of each lifestyle factor could not be estimated. Additional variables such as BMI, smoking status, and comorbidities were considered but not included to maintain model parsimony. A formal directed acyclic graph was not constructed. Given the exploratory nature of this analysis, no statistical correction for multiple comparisons was applied.

Postoperative years were categorized based on previous findings indicating that intestinal function recovers within two years after colorectal anastomosis.^32^ The associations are reported using partial regression coefficients with 95% confidence intervals (CI), and corresponding P values are presented in the tables. Statistical analyses were conducted using R version 4.4.1 (R Foundation for Statistical Computing, Vienna, Austria). The two-sided significance level was set at *P* < 0.05.

Model assumptions were assessed using standard residual diagnostics, including Q–Q plots for normality and visual inspection of residuals versus fitted values for homoscedasticity and linearity. Given the exploratory nature and limited sample size, formal sensitivity analyses were not performed beyond standard model diagnostics, limiting the ability to assess the robustness of the findings. The results should therefore be interpreted with caution.

## Results

Model diagnostics indicated that no major violations of linear regression assumptions (normality of residuals, homoscedasticity, absence of multicollinearity, and lack of influential outliers) were observed.

### Patient characteristics

**Fig. 1:**
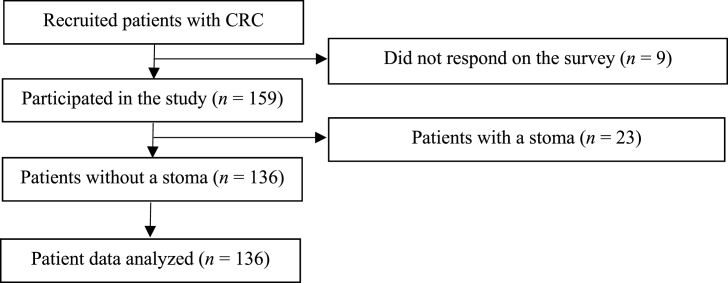
Flowchart of the participant selection process. CRC, colorectal cancer.

Fig. 1 presents the participant flowchart. We recruited 168 patients with CRC, of whom 159 met the eligibility criteria and provided informed consent. Among them, data from 136 patients without a stoma (85.5%) were included in the analysis. All completed questionnaires were collected in person, with a response rate of 100% and no missing data. Table 1 summarizes the participants' characteristics.

**Table 1:** Patient characteristics.

Characteristics	Overall (*n* = 136)	Men (*n* = 68)	Women (*n* = 68)
Age, years, median [IQR]	72.0 [62.0, 77.0]	73.0 [64.0, 78.3]	68.5 [58.0, 76.0]
BMI, kg/m^2^, mean (SD)	22.2 (3.9)	23.1 (3.6)	21.4 (4.0)
Living alone, *n* (%)	50 (36.8)	31 (45.6)	19 (27.9)
Employed, *n* (%)	33 (24.3)	21 (30.9)	12 (17.6)
**TNM-UICC stage, *n* (%)**			
I	27 (19.9)	15 (22.1)	12 (17.6)
II	41 (30.1)	21 (30.9)	20 (29.4)
III	59 (43.4)	29 (42.6)	30 (44.1)
IV	8 (5.9)	2 (2.9)	6 (8.8)
Unknown	1 (0.7)	1 (1.5)	0 (0.0)
**Operative procedure, *n* (%)**			
LAR	58 (42.6)	33 (48.5)	25 (36.8)
HAR	2 (1.5)	1 (1.5)	1 (1.5)
ISR	7 (5.1)	5 (7.4)	2 (2.9)
Colectomy	69 (50.7)	29 (42.6)	40 (58.8)
**Medical history, *n* (%)**			
Diabetes mellitus	4 (2.9)	2 (2.9)	2 (2.9)
Hypertension	14 (10.3)	6 (8.8)	8 (11.8)
Angina pectoris	1 (0.7)	0 (0.0)	1 (1.5)
Respiratory illness	1 (0.7)	0 (0.0)	1 (1.5)
**Chemotherapy,*****n*****(%)**	28 (20.6)	15 (22.1)	13 (19.1)
**Laxative use,*****n*****(%)**	44 (32.4)	14 (20.6)	30 (44.1)
**Tumor location, *n* (%)**			
Rectum	64 (47.1)	37 (54.4)	27 (39.7)
Colon	72 (52.9)	31 (45.6)	41 (60.3)
**Postoperative years, median [IQR]**	1.95 [0.60, 4.00]	1.95 [0.60, 3.68]	1.95 [0.67, 4.03]
**Postoperative years ≥ 2 years,*****n*****(%)**	68 (50.0)	34 (50.0)	34 (50.0)
**Dietary habits, *n* (%)**			
Red meat intake ≥ 500 g/week	38 (27.9)	21 (30.9)	17 (25.0)
Fiber-rich foods intake ≥ 200 g/day	81 (59.6)	42 (61.8)	39 (57.4)
Sugar-sweetened beverage intake ≥ 250 g/day	6 (4.4)	2 (2.9)	4 (5.9)
Alcohol intake ≥ 28 g/day	21 (15.4)	15 (22.1)	6 (8.8)
**Physical activity, *n* (%)**			
Total PA ≥ 300 min/week	44 (32.4)	27 (39.7)	17 (25.0)
Occupational PA ≥ 100 min/week	27 (19.9)	15 (22.1)	12 (17.6)
Transportation PA ≥ 100 min/week	37 (27.2)	22 (32.4)	15 (22.1)
Recreational PA ≥ 100 min/week	35 (25.7)	22 (32.4)	13 (19.1)
Sedentary time ≥ 8 h/day	55 (40.4)	29 (42.6)	26 (38.2)
**Fecal incontinence**			
Wexner score, median [IQR]	6.0 [4.0, 8.0]	5.0 [4.0, 7.0]	6.0 [4.0, 9.0]
Incontinence episodes/day, mean (SD)	1.9 (0.9)	1.9 (0.8)	1.9 (1.0)
Use of incontinence pads, *n* (%)	28 (20.6)	9 (13.2)	19 (27.9)

Data are presented as *n* (%) or mean ± standard deviation, or median [interquartile range] unless otherwise specified.

BMI, body mass index; IQR, interquartile range; SD, standard deviation; TNM-UICC, tumor-node-metastasis staging according to the Union for International Cancer Control; LAR, low anterior resection; HAR, high anterior resection; ISR, intersphincteric resection; PA, physical activity.

### Fecal incontinence

FI was assessed in all 136 patients by using the Wexner score. Five patients (3.7%) had no FI (0 points), whereas 131 (96.3%) had FI (1–20 points). FI severity was further classified as follows: mild (1–4 points) in 43 patients (31.6%), moderate (5–8 points) in 57 patients (41.9%), severe (9–12 points) in 23 patients (16.9%), highly severe (13–16 points) in 7 patients (5.1%), and extremely 7 (17–20 points) in 1 patient (0.7%). The mean number of incontinence episodes was 1.9 ± 0.9 per day.

### Dietary habits and fecal incontinence

Table 2 presents the results of multiple regression analysis examining the association between dietary habits and FI severity. Higher red meat intake (≥ 500 g/week) was associated with lower FI severity (*β* = −1.42; 95% CI, −2.71 to −0.12). The intake of other dietary components showed no statistically significant association with FI severity.

**Table 2:** Associations between dietary habits and fecal incontinence severity.

Dietary variable	Wexner score (*n* = 136), β (95% CI)	*P* value
Red meat ≥ 500 g/week	**−1.42 (−2.71, −0.12)**	**0.034**
Fiber-rich foods ≥ 200 g/day	0.24 (−0.98, 1.45)	0.706
Sugar-sweetened beverages ≥ 250 g/day	2.82 (−0.02, 5.66)	0.054
Alcohol ≥ 28 g/day	0.56 (−1.13, 2.25)	0.518

All models were adjusted for sex, age, chemotherapy use, laxative use, tumor location, and postoperative years.

Bold values indicate statistical significance (*P* < 0.05).

*β*, regression coefficient; CI*,* confidence interval. A negative β value indicates lower fecal incontinence severity.

### Physical activity, sedentary behavior, and fecal incontinence

Table 3 presents the results of the multiple regression analysis examining the association of PA and sedentary behavior with FI severity. No significant associations were observed between PA, including total PA, occupational PA, transportation PA, recreational PA, and FI severity. Prolonged sedentary time (≥ 8 h/day) was associated with higher FI severity (*β* = 1.36; 95% CI, 0.18 to 2.54).

**Table 3:** Associations between physical activity, sedentary behavior, and fecal incontinence severity.

GPAQ variable	Wexner score (*n* = 136), *β* (95% CI)	*P* value
Total PA ≥ 300 min/week	0.61 (−0.68, 1.90)	0.356
Occupational PA ≥ 100 min/week	0.54 (−0.94, 2.01)	0.479
Transportation PA ≥ 100 min/week	0.50 (−0.86, 1.86)	0.476
Recreational PA ≥ 100 min/week	−0.10 (−1.47, 1.27)	0.886
Sedentary time ≥ 8 h/day	**1.36 (0.18, 2.54)**	**0.025**

All models were adjusted for sex, age, chemotherapy use, laxative use, tumor location, and postoperative years.

Bold values indicate statistical significance (*P* < 0.05).

GPAQ, Global Physical Activity Questionnaire; PA, physical activity; *β*, regression coefficient; CI, confidence interval; h*,* hour. A positive β value indicates higher fecal incontinence severity.

## Discussion

### Main findings

We examined the associations between dietary habits, PA, and FI severity in patients with CRC after MIS.

In this exploratory cross-sectional study, higher red meat intake was associated with lower FI severity, whereas prolonged sedentary time (≥ 8 h/day) was associated with higher FI severity; other PA-related variables showed no significant associations.

Although causal inferences cannot be made due to the cross-sectional design, these findings suggest that dietary habits and sedentary behavior may be relevant to the understanding of FI severity in this population. Given the exploratory nature of this study and the relatively limited sample size, the analyses may have been underpowered to detect modest associations. Therefore, non-significant findings should not be interpreted as evidence of no association, but rather as inconclusive findings. Patients with higher red meat intake (≥ 500 g/week) showed a 1.42-point lower Wexner score compared with those with lower intake, indicating an inverse association between red meat intake and FI severity. Although this difference did not exceed the established minimal clinically important difference (MCID) of −2 to −3 points, prior research suggests that even a 1–2 point difference may have clinical relevance.^31^ However, this difference should not be interpreted as definitively clinically meaningful. Our findings align with previous studies showing improved bowel function in patients with higher livestock meat intake after sphincter-preserving surgery^33^ and fewer bowel movements among meat-eaters than vegetarians.^34^ Possible explanations include red meat's lack of dietary fiber, which may slow intestinal transit and firm stool, thus reducing urgency and leakage in some patients.^35^ In contrast, excessive red meat intake is a known risk factor for CRC.^24^ Therefore, our results should not be interpreted as advocating increased red meat consumption, but rather as highlighting the potential role of stool consistency in relation to FI severity. Although our regression models adjusted for major available covariates, residual confounding remains possible. Several lifestyle-related factors, including BMI, smoking status, alcohol consumption, and vegetable intake, were measured in this study but were not included in the final models to maintain model stability. As these factors may be associated with both red meat consumption and bowel function, their exclusion should be considered when interpreting the observed association.

Our results differ from those of Greenberg et al., who found that greater adherence to the American Cancer Society guidelines, including lower red meat intake, was associated with better bowel function.^10^ This discrepancy may be due to differences in scope and outcomes: Greenberg et al. examined overall dietary patterns and general bowel function, while our study focused specifically on red meat intake and FI severity. Moreover, differences in treatment modalities, tumor location, and use of neoadjuvant/adjuvant therapies may have contributed to the differing outcomes.

The observed 1.36-point difference in Wexner scores between patients with < 8 h and ≥ 8 h of sedentary time per day did not exceed the MCID. However, Bols et al.^30^ reported that even a one-point change in the Wexner score may be associated with noticeable differences in FI-related QoL. Prolonged sitting may alter pelvic alignment and reduce pelvic floor muscle activation, as changes in sitting posture have been shown to affect pelvic floor muscle activity.^36^ Previous cross-sectional studies have also reported associations between sedentary behavior and FI among individuals with type 2 diabetes and between long sedentary time and urinary incontinence in the general population.^37^^,^^38^ These findings suggest that prolonged sedentary behavior may be an important lifestyle correlate of FI severity.

Therefore, reverse causation should also be considered. Patients with severe FI may avoid consuming red meat or sugar-sweetened beverages if they believe these worsen their symptoms, potentially creating an apparent association between higher intake and lower FI severity. Similarly, patients with severe FI may limit going out and spend more time sitting at home, resulting in longer sedentary periods. These possibilities highlight the importance of cautious interpretation of our findings.

### Implications for nursing practice and research

To better address potential reverse causation, future research should adopt longitudinal or interventional designs to clarify the directionality of observed associations. Specifically, longitudinal studies should measure baseline lifestyle factors, such as dietary intake and PA levels, and follow patients over time to observe the onset or progression of FI. In addition, interventional studies targeting modifiable exposures, such as increased dietary fiber intake or PA, would allow evaluation of whether these changes lead to clinically meaningful improvements in FI severity. These approaches would help clarify causal relationships and guide evidence-based lifestyle interventions for patients with CRC following MIS.

### Strengths and limitations

A major strength of this study is that, to our knowledge, it is the first to examine the associations between FI severity, as measured by the Wexner score, and dietary habits, PA, and sedentary behavior in patients with CRC after minimally invasive surgery. These findings may contribute to the development of lifestyle-based approaches to managing FI. Another strength is the high completeness of the dataset, achieved through on-site data collection and in-person confirmation by trained research staff, which minimized missing data and allowed clarification of unclear responses.

This study has several limitations. First, the relatively small sample size and the exploratory cross-sectional design limited statistical power, reduced generalizability, and precluded causal inference. This may have reduced the ability to detect modest associations; therefore, non-significant findings should be interpreted with caution and should not be taken as evidence of no association.

Second, continuous variables were categorized using predefined cutoffs based on established guidelines. This approach, while adopted to enhance clinical interpretability and model stability, may have resulted in information loss, obscured potential dose-response relationships, and reduced statistical power.

Third, lifestyle-related exposures were analyzed in separate regression models. Although this approach was used to reduce the risk of overfitting, it may have limited the ability to fully account for confounding between these variables, and residual confounding cannot be excluded.

Fourth, this study was conducted at a single center and included patients attending follow-up care, which may have introduced selection related to healthcare-seeking behavior and referral patterns.

Fifth, dietary habits and FI were assessed using brief self-administered questionnaires that required participants to estimate their usual intake in grams. Furthermore, dietary fiber was assessed based on food intake rather than actual fiber quantity.^39^ FI severity and PA were also self-reported and may have been affected by recall or social desirability bias.

Finally, because this study was exploratory in nature and aimed to identify overall trends rather than to confirm causal relationships, formal sensitivity analyses were not conducted. This may limit the ability to assess the robustness of the findings.

Future studies with larger sample sizes, validated measurement tools, and longitudinal or interventional designs are needed to confirm these findings and clarify causal relationships.

## Conclusions

Our findings indicate that red meat intake and sedentary behavior were associated with FI severity among CRC survivors after MIS. Specifically, higher red meat intake was associated with lower FI severity, whereas prolonged sedentary time was associated with higher FI severity. These findings should be interpreted cautiously, as the exploratory cross-sectional design does not allow for causal inference. Rather than advocating changes in red meat consumption, the observed associations should be regarded as hypothesis-generating observations regarding lifestyle correlates of FI severity. Further longitudinal and interventional studies are required to clarify the temporal direction and clinical significance of these associations.

## CRediT authorship contribution statement

Conceptualization: Hiromi Nakagawa and Hiroyuki Sasai; Methodology: Hiromi Nakagawa and Hiroyuki Sasai; Software: Hiromi Nakagawa, Sho Hatanaka, and Hiroyuki Sasai; Validation: Hiromi Nakagawa and Hiroyuki Sasai; Formal analysis: Hiromi Nakagawa, Sho Hatanaka, and Hiroyuki Sasai; Investigation: Hiromi Nakagawa and Nobuhisa Matsuhashi; Resources: Hiromi Nakagawa; Data curation: Hiromi Nakagawa; Writing - original draft preparation: Hiromi Nakagawa and Hiroyuki Sasai; Writing - review and editing: Hiromi Nakagawa and Hiroyuki Sasai; Visualization: Hiromi Nakagawa and Hiroyuki Sasai; Supervision and Project administration: Hiromi Nakagawa and Hiroyuki Sasai; Funding acquisition: Hiromi Nakagawa. All authors have read and approved the final manuscript.

## Ethics statement

The study was approved by the Institutional Review Board of Gifu University (No. 2023-207, Approval date: December 21, 2023) and was conducted in accordance with the 1964 Helsinki Declaration and its later amendments or comparable ethical standards. All participants provided written informed consent.

## Data availability statement

The data that support the findings of this study are not publicly available due to privacy and ethical restrictions but are available from the corresponding author, HN, upon reasonable request.

## Declaration of generative AI and AI-assisted technologies in the writing process

During the preparation of this manuscript, the authors used generative AI tools (ChatGPT) solely for language editing and improving clarity. After using these tools, the authors carefully reviewed and revised all content and take full responsibility for the scientific integrity and accuracy of the manuscript.

## Funding

This study was funded by the 10.13039/501100001691Japan Society for the Promotion of Science (JSPS) KAKENHI (Grant No. JP 20K10728 and 24K13719). The funders had no role in considering the study design or in the collection, analysis, interpretation of data, writing of the report, or decision to submit the article for publication.

## Declaration of competing interest

The authors have no competing interests to declare that are relevant to the content of this article.

## References

[1] Ferlay J., Ervik M., Lam F. (2022). https://gco.iarc.who.int/today.

[2] Sultan A.H., Monga A., Lee J. (2017). An International Urogynecological Association/International Continence Society joint report on the terminology for female anorectal dysfunction. Neurourol Urodyn.

[3] Farouk R., Duthie G.S., Lee P.W.R., Monson J.R.T. (1998). Endosonographic evidence of injury to the internal anal sphincter after low anterior resection. Dis Colon Rectum.

[4] Verkuijl S.J., Jonker J.E., Trzpis M., Burgerhof J.G.M., Broens P.M.A., Furnée E.J.B. (2021). Functional outcomes of surgery for colon cancer: a systematic review and meta-analysis. Eur J Surg Oncol.

[5] Al Rashid F., Liberman A.S., Charlebois P. (2022). The impact of bowel dysfunction on health-related quality of life after rectal cancer surgery: a systematic review. Tech Coloproctol.

[6] Nguyen T.H., Chokshi R.V. (2020). Low anterior resection syndrome. Curr Gastroenterol Rep.

[7] Sun V., Thomson C.A., Crane T.E. (2024). Baseline characteristics and recruitment for SWOG S1820: altering intake, managing bowel symptoms in survivors of rectal cancer (AIMS-RC). Support Care Cancer.

[8] Pun M.Y., Leung P.H., Chan T.C. (2024). The effectiveness of physiotherapy interventions on fecal incontinence and quality of life following colorectal surgery: a systematic review and meta-analysis of randomized controlled trials. Support Care Cancer.

[9] Staller K., Song M., Grodstein F. (2018). Increased long-term intake is associated with a decreased risk of fecal incontinence in older women. Gastroenterology.

[10] Greenberg A.L., Tolstykh I.V., Van Loon K. (2023). Association between adherence to the American Cancer Society nutrition and physical activity guidelines and stool frequency among colon cancer survivors: a cohort study. J Cancer Surviv.

[11] Smith K.S., Gudenkauf L.M., Hoogland A.I. (2024). Associations between dietary patterns and quality of life in a longitudinal cohort of colorectal cancer survivors. Nutrients.

[12] Temple L.K., Bacik J., Savatta S.G. (2005). The development of a validated instrument to evaluate bowel function after sphincter-preserving surgery for rectal cancer. Dis Colon Rectum.

[13] Liu W., Xia H.O. (2024). The impact of dietary factors on the relief of bowel dysfunction among patients with rectal cancer after the sphincter-saving surgery: a prospective cohort study. J Gastrointest Cancer.

[14] DiNicolantonio J.J., Lucan S.C. (2015). Is fructose malabsorption a cause of irritable bowel syndrome?. Med Hypotheses.

[15] Maccioni L., Fu Y., Horsmans Y. (2023). Alcohol-associated bowel disease: new insights into pathogenesis. eGastroenterology.

[16] Gonçalves dos Santos M., Mazo G.Z., de Avelar N.C., Cidade B.S., Mondardo B.O., Virtuoso J.F. (2024). Symptoms of pelvic floor disorders and physical fitness: a comparison between active and sedentary older women—a cross-sectional study. Geriatr Nurs.

[17] Kenkhuis M.F., Klingestijn M., Fanshawe A.M. (2023). Longitudinal associations of sedentary behavior and physical activity with body composition in colorectal cancer survivors up to 2 years post treatment. J Cancer Res Clin Oncol.

[18] Viskochil R.H., Lin T., Gigic B. (2025). Sedentary behavior and physical activity one year after colorectal cancer diagnosis: results from the ColoCare Study. J Cancer Surviv.

[19] Dexter E., Walshaw J., Wynn H. (2024). Faecal incontinence—a comprehensive review. Front Surg.

[20] Rao S.S.C. (2004). Pathophysiology of adult fecal incontinence. Gastroenterology.

[21] Ekici E., Özkeskin M., Özden F. (2023). The effect of exercise on patients with colorectal cancer surgery: a systematic review. Surg Pract Sci.

[22] Grosek J., Ales Kosir J., Sever P., Erculj V., Tomazic A. (2021). Robotic versus laparoscopic surgery for colorectal cancer: a case-control study. Radiol Oncol.

[23] Rock C.L., Thomson C.A., Sullivan K.R. (2022). American Cancer Society nutrition and physical activity guideline for cancer survivors. CA Cancer J Clin.

[24] Clinton S.K., Giovannucci E.L., Hursting S.D. (2020). The World Cancer Research Fund/American Institute for Cancer Research Third Expert Report on diet, nutrition, physical activity, and cancer: impact and future directions. J Nutr.

[25] Bull F.C., Maslin T.S., Armstrong T. (2009). Global Physical Activity Questionnaire (GPAQ): nine country reliability and validity study. J Phys Act Health.

[26] World Health Organization (2020). https://www.who.int/publications/i/item/9789240014886.

[27] Bourke L., Homer K.E., Thaha M.A. (2014). Interventions to improve exercise behaviour in sedentary people living with and beyond cancer: a systematic review. Br J Cancer.

[28] Ekelund U., Steene-Johannessen J., Brown W.J. (2016). Does physical activity attenuate, or even eliminate, the detrimental association of sitting time with mortality? A harmonised meta-analysis of data from more than 1 million men and women. Lancet.

[29] Bols E.M.J., Hendriks H.J.M., Berghmans L.C.M., Baeten C.G.M.I., De Bie R.A. (2013). Responsiveness and interpretability of incontinence severity scores and FIQL in patients with fecal incontinence: a secondary analysis from a randomized controlled trial. Int Urogynecol J.

[30] Jorge J.M., Wexner S.D. (1993). Etiology and management of fecal incontinence. Dis Colon Rectum.

[31] Croese A.D., Lonie J.M., Trollope A.F., Vangaveti V.N., Ho Y.H. (2018). A meta-analysis of the prevalence of low anterior resection syndrome and systematic review of risk factors. Int J Surg.

[32] Ho Y.H., Com W. (2006). Techniques for restoring bowel continuity and function after rectal cancer surgery. World J Gastroenterol.

[33] Liu W., Xia H.O. (2022). The association between dietary intake and improvement of LARS among rectal cancer patients after sphincter-saving surgery—a descriptive cohort study. Front Surg.

[34] Sanjoaquin M.A., Appleby P.N., Spencer E.A., Key T.J. (2004). Nutrition and lifestyle in relation to bowel movement frequency: a cross-sectional study of 20,630 men and women in EPIC-Oxford. Public Health Nutr.

[35] Heinritz S.N., Weiss E., Eklund M. (2016). Intestinal microbiota and microbial metabolites are changed in a pig model fed a high-fat/low-fiber or a low-fat/high-fiber diet. PLoS One.

[36] Sapsford R.R., Richardson C.A., Maher C.F., Hodges P.W. (2008). Pelvic floor muscle activity in different sitting postures in continent and incontinent women. Arch Phys Med Rehabil.

[37] Li L.C., Liang L.M., Ji H.Y. (2024). Exploring the association between type 2 diabetes and fecal incontinence in American adults: insights from a large cross-sectional study. Int J Colorectal Dis.

[38] Di X., Yuan C., Xiang L., Wang G., Liao B. (2024). Association between sitting time and urinary incontinence in the US population: data from the National Health and Nutrition Examination Survey (NHANES) 2007 to 2018. Heliyon.

[39] U.S. Department of Agriculture, U.S. Department of Health and Human Services (2025). https://www.dietaryguidelines.gov.

